# Toward a general evolutionary theory of oncogenesis

**DOI:** 10.1111/eva.12023

**Published:** 2012-12-05

**Authors:** Paul W Ewald, Holly A Swain Ewald

**Affiliations:** Department of Biology, University of LouisvilleLouisville, KY, USA

**Keywords:** adaptation, cancer, evolutionary medicine, host parasite interactions, oncogenesis, tumor viruses

## Abstract

We propose an evolutionary framework, the barrier theory of cancer, which is based on the distinction between barriers to oncogenesis and restraints. Barriers are defined as mechanisms that prevent oncogenesis. Restraints, which are more numerous, inhibit but do not prevent oncogenesis. Processes that compromise barriers are essential causes of cancer; those that interfere with restraints are exacerbating causes. The barrier theory is built upon the three evolutionary processes involved in oncogenesis: natural selection acting on multicellular organisms to mold barriers and restraints, natural selection acting on infectious organisms to abrogate these protective mechanisms, and oncogenic selection which is responsible for the evolution of normal cells into cancerous cells. The barrier theory is presented as a first step toward the development of a general evolutionary theory of cancer. Its attributes and implications for intervention are compared with those of other major conceptual frameworks for understanding cancer: the clonal diversification model, the stem cell theory and the hallmarks of cancer. The barrier theory emphasizes the practical value of distinguishing between essential and exacerbating causes. It also stresses the importance of determining the scope of infectious causation of cancer, because individual pathogens can be responsible for multiple essential causes in infected cells.

## Introduction: evolutionary processes in oncogenesis

The history of medicine informs us that understanding the causes of disease is the best approach to prevention and cure. The history of biology tells us that a thorough understanding of life processes requires an integration of evolutionary and mechanistic explanations. Although studies of cancer have gradually incorporated evolutionary perspectives (see Aktipis et al. 2011), evolutionary explanations are often inserted into discussions of cancer rather than being used as a conceptual framework.

The most widely appreciated application of evolutionary logic to the study of cancer involves oncogenesis itself, which we define as the process by which normal cells acquire the characteristics of cancer cells. This definition encompasses the changes that occur prior to and after the threshold of cancer is reached. Normal cells evolve into cancerous cells partly as a result of selection acting on variation generated by genetic mutations that arise within multicellular organisms, which in turn result from exposure to mutagens, such as radiation and mutagenic chemicals, and errors in DNA replication. Over the past few decades, it has become apparent that a staggering number of mutations occur during oncogenesis and that the composition of these mutations usually varies greatly from cancer to cancer even among cancers of the same type (e.g., Chapman et al. 2011; Nik-Zainal et al. 2012). It has also become apparent that mutations vary greatly in their significance for oncogenesis (e.g., Meyerson et al. 2010), that the microenvironments in which a cell resides can have important influences (Mueller and Fusenig 2004; Kim et al. 2011; Gatenby and Gillies 2008), and that infectious agents often play major roles (Bouvard et al. 2009; zur Hausen 2010; Ewald and Swain Ewald 2012).

These realizations challenge the simplistic idea that cancer could be understood by identifying a small number of key mutations. Rather, they emphasize the need for a broad and deep evolutionary theory of cancer that can provide a coherent framework for the vast amounts of information that has been and will be discovered. Issues that need to be incorporated in such a theory have been discussed (Greaves 2000, 2002; Merlo et al. 2006; DeGregori 2011; Gatenby and Gillies 2008; Greaves and Maley 2012). Here, we work toward the goal of a simple yet comprehensive evolutionary theory of cancer by presenting a conceptual framework that integrates the key aspects of oncogenesis. We then assess the implications of this understanding for prevention and treatment.

Starting from first principles, we recognize that there are three evolutionary processes that are critically involved in oncogenesis. As mentioned above, the most obvious of these processes is the evolution of normal cells into cancerous cells and the evolution of cancerous cells thereafter (Nowell 1976; Heppner and Miller 1998; Crespi and Summers 2005; Merlo et al. 2006; Gillies et al. 2012). A second evolutionary process has generated characteristics of multicellular organisms that reduce the risk of cancer. The most absolute of these anticancer characteristics are barriers, such as cell cycle arrest, that prevent oncogenesis by prohibiting the replication of cells. It is now becoming clear that natural selection enhances barriers to cancer in proportion to the organisms need for these barriers – large, long-lived organisms have evolved enhanced anticancer adaptations relative to small, short-lived organisms (Seluanov et al. 2008; Caulin and Maley 2011; DeGregori 2011). A third evolutionary process involves adaptations of infectious organisms that enhance their tendency to compromise host cell defenses against cancer (Ewald 2009; Ewald and Swain Ewald 2012).

The evolution of anticancer adaptations and oncogenic characteristics of infectious organisms invoke natural selection as Darwin envisioned it; in both cases, natural selection can favor heritable changes indefinitely through advantages they bestow on survival and reproduction of organisms (i.e., through increases in evolutionary fitness). Cells within multicellular organisms restrict their own survival and reproduction because this regulation increases the passing on of their genetic instructions across generations. Similarly, infectious agents evolve characteristics that exploit their hosts, to the extent that this exploitation increases the passing on of genetic instructions for these characteristics indefinitely over cycles of transmission between hosts.

In contrast, the selective process that drives the evolution of normal cells into cancer cells is not open-ended. With rare exceptions, cancer cells are not transmissible from one individual to another. They therefore are not shaped by the unending cumulative change that is inherent to natural selection, narrowly defined. Recognizing this difference, we use the term oncogenic selection for the selection that favors evolution of cancer cells from normal cells. The difference between oncogenic selection and natural selection leads to a fundamental difference in evolutionary outcomes of selection. Oncogenic selection is largely a destructive process through which cells lose regulatory mechanisms that have been refined by natural selection to keep the cells functioning in the genetic interest of the organism. The two processes are coevolutionarily interconnected: natural selection favors the evolution of characteristics that inhibit oncogenesis, while oncogenic selection favors their abrogation.

We draw together these aspects of oncogenesis into a *barrier theory of cancer*, which is structured by an understanding of how evolutionary processes simultaneously shape host defenses against cancer and the abrogation of these defenses by mutation and infection. We compare and contrast this barrier theory with other current conceptual frameworks for understanding cancer and options for prevention and treatment.

## Barriers to cancer

An evolutionary perspective on cancer must integrate these processes of natural and oncogenic selection. Acting on the genetic make-up of multicellular organisms, natural selection has generated *barriers* to cancer, which we define as mechanisms that block progression to cancer. Current knowledge allows five categories of adaptations to be classified as barriers to metastatic cancer: cell cycle arrest, apoptosis, caps on the total number of future cell divisions, cell adhesion, and asymmetric cell division (i.e., a stem cell division that generates one stem cell and one cell destined to differentiate during subsequent proliferation). We distinguish such barriers from *restraints*, which slow or inhibit the progression to cancer. An example of a restraint is the regulation of the rate of division of a dividing cell. This restraint does not prevent cancer, but it may retard oncogenesis and generate a cancer cell that is less damaging.

The collection of barriers that are in place in a cell depends on the cell type. Some mechanisms function as barriers for almost all normal cells. Cell cycle arrest, for example, is a pervasive barrier, because normal cells cannot evolve into cancerous cells if cell cycle arrest is in place.

Apoptosis is a barrier to cancer in most cell types because a dead cell cannot be a cancerous cell. But in some cell types, intact apoptotic mechanisms can still permit oncogenesis if the cell has a high threshold for initiation of apoptosis (Savage et al. 2009). Natural selection may have favored a high threshold in cells that must have elevated rates of genetic alterations as part of their normal functioning (e.g., B lymphocytes), so that they will not be destroyed in response to these genetic alterations (Savage et al. 2009, Ewald and Swain Ewald 2012).

Regulation of telomerase can be an effective barrier to oncogenesis when telomeres are short, by restricting the number of cell divisions that can occur. But regulation of telomerase is not always a barrier to cancer. Early in life, when long telomeres permit a large number of future divisions, oncogenesis can occur even if telomerase is regulated. This situation apparently applies to some childhood cancers, such as retinoblastoma (Gupta et al. 1996).

Telomerase regulation is not a barrier to oncogenesis for stem cells, which constitutively express telomerase. Undifferentiated stem cells, however, generally have a different barrier in place: asymmetric cell division. When cell division occurs asymmetrically, stem cell cancer is blocked because stem cell replication is restricted to replacement.

What qualifies as a barrier can also depend on the criteria that one uses for cancer, which in turn can depend on aspects of cancer that are critical to health. For cancers in which metastasis is a critical characteristic, cell adhesion is a barrier because it can prevent metastatic cancer. But if the cancer is not metastatic or if metastasis is not considered to be an essential aspect of the cancer (as is the case with retinoblastoma), cell adhesion is not a barrier. A twist on this theme involves cancers of cells that are not normally restricted by cell adhesion. In leukemia, for example, cell adherence is not a barrier because leukocytes are freely circulating; however, the induction of cell adhesion in lymphocytes can be an exacerbating influence by allowing circulating lymphocytes to develop lymphomas in solid tissues.

## Essential versus exacerbating causes of oncogenesis

Distinguishing barriers from restraints allows for the differentiation between essential and exacerbating causes of oncogenesis. This distinction is of practical importance because preventing an essential cause will prevent the cancer (by preserving the barrier to oncogenesis). Preventing an exacerbating cause may reduce the damage from the cancer or may contribute to prevention or an effective therapy, but will not by itself prevent or cure the cancer.

Essential causes of oncogenesis may vary among cell types. To illustrate this variation, we consider four categories of cells below and summarize our points in Table 1.

**Table 1:** The cellular context of essential causes of oncogenesis. Abrogation of a barrier to oncogenesis is, by definition, an essential cause of cancer, but only if the barrier is actively preventing oncogenesis in the normal cell of a given type. A ‘yes’ indicates that abrogation of the barrier is an essential cause of a cancer of the designated cell type

	Must barrier be abrogated?				
	
Cell type category	Cell cycle arrest	Apoptosis	Telomerase regulation	Cell adhesion	Asymmetric division
B & T lymphocytes	Yes	No	Yes	No*	No
Retinoblasts	Yes	No	No	No	No?
Colonic stem cells	Yes	Yes	No	Yes	Yes
Cervical epithelial cells	Yes	Yes	Yes	Yes	No

* If the cancer originates in nonadherent lymphocytes.

At one end of the spectrum are retinoblasts, which exemplify cell types with few barriers. These slightly differentiated stem cells give rise to retinal cells of the eye during normal development and retinoblastoma during oncogenesis. Mutations in the retinoblastoma gene (which codes for a protein that enforces cell cycle arrest) are considered essential for the development of retinoblastomas, indicating that intact cell cycle arrest meets the definition of a barrier for retinoblastoma, and function destroying mutations in the retinoblastoma gene is an essential cause of retinoblastoma.

As mentioned above, telomerase is often absent in retinoblastoma cells (Gupta et al. 1996). The breaking of repression of telomerase synthesis by a function destroying mutation is therefore not an essential cause of retinoblastoma. Similarly, abrogation of cell adhesion is not an essential cause of retinoblastoma, because nonmetastatic disease is considered part of the spectrum of retinoblastoma. Asymmetric division of stem cells is apparently not a characteristic of retinoblasts because the partially differentiated state of the retinoblasts has apparently already shifted them to symmetrical divisions. Destruction of the mechanism for asymmetric division is therefore not an essential cause of retinoblastoma.

Retinoblastoma cells are refractory to apoptosis, but research has not implicated mutations (Poulaki et al. 2005). It appears instead that apoptotic genes are epigenetically silenced by methylation (Poulaki et al. 2005). Abrogation of apoptosis is therefore not an essential cause of retinoblastoma. The reduced tendency to undergo apoptosis might be an adaptation that reduces cell death in response to mutations induced by ultraviolet light (e.g., through UV activation of antiapoptotic pathways; Xia et al. 1995; Glotin et al. 2006; Roduit and Schorderet 2008; Mendes et al. 2009). A low apoptotic threshold for retinal cells may be more detrimental to fitness than the presence of mutations, which generally pose little risk for oncogenesis because the cells soon become terminally specialized. The tendency for retinoblastoma to be limited to the first few years of life, suggests that one or more other barriers are put in place after this time, which virtually eliminate the risk of retinoblastoma.

B and T lymphocytes are partially differentiated cells that must be able to replicate to high numbers under normal conditions but are held in check by regulation of telomerase (Barsov 2011). Assays confirming the ubiquitous presence of telomerase in early stages of lymphocyte oncogenesis (Wu et al. 1999) indicate that disruption of telomerase regulation is an essential cause. It appears that apoptotic mechanisms generally remain intact in lymphomas (Savage et al. 2009). When they remain, functional abrogation of apoptosis is not an essential cause. Cell cycle arrest mechanisms need to be functioning in lymphocytes to curtail their proliferation and maintain memory cell state. Asymmetric division is not in place because lymphocytes need to divide symmetrically to proliferate to large numbers during immunological responses. Cell adhesion does not need to be abrogated to permit metastasis if the cancer originates in nonadherent cells.

Colon crypt stem cells illustrate dependence of oncogenesis on abrogation of asymmetric division. Colon crypt stem cells divide asymmetrically to yield one cell that will replace the stem cell and another that continues to divide symmetrically to generate the epithelial cells that line the colon. Asymmetric cell division is enforced by the adenomatis polyposis coli (APC) protein through its effects on mitotic spindle asymmetry (Yamashita et al. 2003; Quyn et al. 2010). Mutations in the gene that codes for APC are extremely common in colon cancer, and individuals with inherited, nonfunctional APC almost always develop colon cancer by middle age (Markowitz and Bertagnolli 2009). These findings suggest that asymmetric division is a barrier to stem cell-derived colon cancer. Regulation of telomerase is presumably not a barrier to oncogenesis for such stem cells because their stem cell identity implies the presence of telomerase. Cell adhesion would, however, need to be compromised to permit metastatic spread, as would mechanisms for inducing apoptosis and cell cycle arrest.

Cervical epithelial cells being in the post-stem cell phase of proliferation would not have in place the asymmetric division barrier, but would have the other four. Cervical epithelial cells therefore are characterized by a large number of in place barriers and illustrate the opposite side of the barrier spectrum from retinoblasts.

## Integrating infection

Any general theory of oncogenesis must incorporate the role of infection. Current estimates of the proportion of human cancer known to be caused by infectious organisms (subcellular, cellular, and multicellular) range from 16% to just over 20% (zur Hausen 2008; de Martel et al. 2012). The actual pervasiveness of infectious causation of cancer may be much greater because infectious causation has not been adequately evaluated for most human cancers and can be ruled out for only a very small portion. Bouvard et al. (2009) provide a summary of accepted infectious causes of human cancers, as of 2009.

Infectious organisms may be exacerbating causes of cancer if they enhance the mutation-driven process of oncogenesis, for example, by increasing the presence of mutagenic molecules or pro-proliferative signals during inflammation (Moss and Blaser 2005). They may, however, act as essential causes if they compromise barriers to cancer. When a virus has the capability of replicating its genome in concert with replication of its host cell, it can increase its own fitness with low exposure to immunological attack by enhancing the survival and reproduction of the host cell. Viruses can acquire this capacity by abrogating barriers to cancer, particularly cell cycle arrest, apoptosis, telomerase regulation and cell adhesion (Ewald and Swain Ewald 2012). Breaking cell cycle arrest allows the viral genome to replicate. By allowing synthesis of telomerase, the viral genome can remove the limit on the total amount of this replication. By blocking apoptosis, a virus can reduce the chance that it will be destroyed in response to cellular detection of its presence. By reducing cell adhesion, it can seed new locations in the body. Several cancer-causing viruses of humans have been sufficiently well investigated to evaluate whether they compromise these barriers: Epstein Barr Virus, Kaposi's Sarcoma-associated herpes virus, Hepatitis B virus, Hepatitis C virus, Human T-cell lymphotropic virus type 1, and human papillomavirus. Each of these viruses compromises all four barriers (Ewald and Swain Ewald 2012). This simultaneous compromising of four barriers to cancer is important for oncogenesis because it allows for the generation of large populations of infected, dividing cells that are pushed toward the brink of cancer. Oncogenic mutations that are highly improbable in a small population of dysregulated cells can become much more probable in a large population.

These oncogenic effects of infection contrast with the difficulties of mutation-driven oncogenesis in the absence of infection. The probability of an oncogenic mutation is vanishingly small for any particular cell. If several barriers are in place, an uninfected cell must acquire a specific set of mutations under constraints imposed by those barriers that are still intact (e.g., apoptosis and cell cycle arrest in response to mutation or restrictions of the total number of divisions imposed by regulation of telomerase) without acquiring the vastly more common mutations that make the cells unviable.

It is noteworthy that the cancer that seems most thoroughly explained by mutations without any joint role for infections, retinoblastoma, is the cancer that seems to have the fewest barriers in place in the cells of origin (Table 1). Even so, it occurs in only one of every 15 000 individuals. It is thought that virtually all individuals who have mutations in both copies of the retinoblastoma gene during the first few years of life will develop retinoblastoma. The absence of retinoblastoma at later ages indicates that one or more barriers to retinoblastoma are put in place after these early years.

## The extended phenotype and the microenvironment

Oncogenesis needs to be considered in its microenvironmental context (Mueller and Fusenig 2004; DeGregori 2011; Kim et al. 2011; Gatenby and Gillies 2008). The microenvironment of cancerous and precancerous cells includes genetically normal cells and extracellular constituents, such as signaling molecules and proteolytic enzymes, which can enhance cell growth and contribute to metastasis. Normal fibroblasts, for example, can enhance oncogenesis by release of proliferative signals (Bhowmick et al. 2004; Schauer et al. 2011) Extracellular metalloproteases contribute to metastasis by degrading cell adhesion molecules and may influence proliferation, apoptosis, and angiogenesis (Egeblad and Werb 2002; Bourboulia and Stetler-Stevenson 2010). Microenvironmental influences also include development of genetically normal tissues in ways that foster oncogenesis. Growth of blood vessels at the location of a developing tumor, for example, involves responses of cells to microenvironmental states, such as hypoxia (Kerbel 2000). Angiogenesis may help sustain tumor development even though the cells of the blood vessels are not themselves cancerous or precancerous.

Principles of natural selection suggest that biochemical pathways within and between cells will be so intricately interconnected that alteration of one component will tend to have numerous, complex effects (Ewald and Swain Ewald 2011). We can therefore expect the documentation of microenvironmental influences to continue to increase in number and complexity. Considerations of microenvironmental influences, like mutational analyses, could rapidly become overwhelmed by complexity and detail.

In an attempt to deal with this problem, researchers have made arguments based on analogies with organismal development (Egeblad et al. 2010). Cellular activity in organismal development, however, is tuned by natural selection to function cooperatively with the rest of the organism. In tumor development, oncogenic selection breaks down this tuning through events, such as mutations or infections, that free cells from the regulatory restrictions imposed by natural selection. This difference between effects of oncogenic selection and natural selection together with the limited time frame for oncogenic selection suggest that the large variety of different cells intimately associated with a tumor will not serve functions for the tumor in the same way that cells in a body serve functions for the body. Rather, alterations of noncancerous cells in the microenvironment of tumors will tend to occur in response to the cells that have been genetically altered during oncogenesis.

These considerations emphasize the need for an evolutionary framework that is based on mechanisms of selection rather than analogy and that clarifies the significance of microenvironmental alterations by distinguishing fundamental processes from secondary ones. The building block for any evolutionary theory of cancer is selection at the genetic level, just as it is for evolutionary theory in general. For oncogenic selection, the focus is on the genetic variants that become more common as a result of increased multiplication or survival of cells. Gatenby and Gillies (2008) provide a conceptual framework to clarify how precancerous cells evolve to better exploit the microenvironmental changes that are associated with oncogenesis. Using the concept of barriers to cancer much more narrowly, we develop this theme by anchoring microenvironmental interactions with the genetic changes that are ultimately responsible for oncogenesis.

The difficulty in conceptually organizing the tremendous number of microenvironmental interactions among normal cells and cells that are genetically modified during oncogenesis is analogous to the difficulty in organizing the countless interactions between organisms and their environments. Dawkins's (1983) concept of the extended phenotype clarified the action of selection on organisms in their natural environments. We propose that this concept brings similar clarity to the action of oncogenic selection on cells in their microenvironments.

Defined as the effects of a genetic variant on its environment, the extended phenotype extends the concept of phenotype to include any characteristic that is influenced by a genetic instruction, even if the characteristic is not part of the living entity. The environment in this case extends to any aspect of the outside world (Dawkins 1983). A bird's nest is part of the extended phenotype of the genetic instructions that led to the nest-building behavior. Genetic instructions in the trematode, *Dicrocoelium dendriticum*, cause its ant host to climb a blade of grass and be eaten by the next host in the trematode's life cycle (a sheep); this behavioral change in the ant is part of the extended phenotype of genetic instructions in the trematode.

Similarly, the microenvironmental changes that arise during oncogenesis are direct or indirect effects of the genetic changes in the cells that contribute to oncogenesis. The extended phenotype concept emphasizes that advantages in oncogenic selection could be intrinsic to the cell programming or could arise through interactions with the cell's microenvironment. Changes in genetically normal cells in the microenvironment may contribute to cancer development, but unlike the genetic variations that lead a cell down the path to cancer, the contributions of normal cells in the microenvironment are not fundamental drivers of oncogenesis. Nor should their contribution be presumed to be analogous to the cooperation of cells during development of organs or organisms. Rather normal cells in the microenvironment of a cancer cell are part of the biological environment that is available to the cancer-promoting genetic instructions of the cancer cell. When these cells are altered in a way that favors the cancer cell through oncogenic selection, they are like the ant that is altered in a way that favors the trematode through natural selection.

Angiogenesis offers an illustration. It is an adaptation for providing gas exchange and nutrients to tissues that are short on resources when, for example, cell populations grow during development or repair after injury, and in response to low oxygen concentration (Kerbel 2000). The hypoxia induced by the elevated multiplication and metabolism of cancerous and precancerous cells therefore results in an extended phenotype that encompasses angiogenesis of normal cells in the vicinity.

The genetic basis for an alteration of the cancer cell's extended phenotype could be mutations in the cell's genome. Or it could be introduced into the cell, as would occur if the cell is infected by an oncogenic virus. In this case, the extended phenotype of the oncogenic viral genes includes the altered growth of a virally infected cell as well as the modifications of the microenvironment that are induced by the virally infected cell.

Whether the genetic basis of the cancer cell's phenotype is generated by mutation or by infection, the extended phenotype includes effects on extracellular components, as well as on normal, cancerous, and precancerous cells in the microenvironment. The extended phenotype perspective together with the concepts of essential and exacerbating causes provides a frame of reference that can help distinguish the most important processes in oncogenesis from the variety and complexity of microenvironmental changes that are inherent to oncogenesis. Microenvironmental changes that facilitate alterations in cell cycle arrest, apoptosis, cell immortalization, and cell adhesion warrant special attention.

The distinction between essential causes and exacerbating causes directs attention to the microenvironmental characteristics for which intervention is expected to have the most significant effects in preventing or treating cancer. With regard to prevention, oncogenic viruses interfere with a suite of essential causes. The extraordinary success at preventing cancer through vaccination and blocking of viral transmission illustrates the value of preventing aspects of the extended phenotype that arise from essential causes. Although cancer treatment does not provide the high benefit to risk ratio achieved by interventions against infectious causes of cancer, the therapeutic interventions that are most successful tend to target essential causes. Antibiotic treatment of *Helicobacter*-induced stomach cancer provides an example for a cancer caused by infection (Bayerdorffer et al. 1995). Imatinib treatment for chronic myeloid leukemia illustrates the targeting of a protein encoded by a mutated gene, which encodes a fusion protein, BCR-ABL, that instigates both essential and exacerbating causes (Bedi et al. 1994; Wertheim et al. 2002).

The extended phenotype of oncogenic genes can involve exploitations of the microenvironment: effects that enhance the success of the genetic instruction through oncogenic selection (a.k.a. manipulation). Alternatively, the extended phenotype can involve defenses: alterations that protect the individual from damage encoded by the oncogenic genetic instruction. Defenses may result, for example, in selection against particular subclones. A third possibility is side effects: alterations that neither enhance the success of the oncogenic instruction nor protect the host from it (categories adapted from Ewald 1980). As discussed above, angiogenesis is thought to reflect an exploitation which enhances the availability of nutrients to the cancer cell. A study of stromal microenvironments adjacent to breast cancers (Finak et al. 2008) provides an illustration of defenses. Patients with favorable outcomes tended to have adjacent microenvironments characterized by protective immune activity: enhanced killer cells and Th1 cell responses. The adjacent microenvironments of poor responders were characterized by a lack of such immune markers, but instead had characteristics associated with exploitation: hypoxia and angiogenesis.

Considering the randomness of new mutations, we can expect that most mutation-induced microenvironmental changes will be side effects rather than defenses or exploitations. Although oncogenic selection will tend to prune side effects that hinder cell function, its time frame is constrained. This logic suggests that a small proportion of the microenvironmental effects of an oncogenic mutation will enhance the success of the mutated cell. In the case of oncogenic viruses, however, natural selection can mold precision in both viral exploitations of the microenvironment and microenvironmental defenses against the infected cell. We therefore expect that side effects will be less prevalent for those aspects of oncogenesis induced by viral infections than for effects of mutations.

The distinctions between defenses, exploitations, and side effects distinguish microenvironmental characteristics of the extended phenotype for which intervention is contra-indicated (i.e., defenses) from microenvironmental changes for which intervention is beneficial (i.e., exploitations and some side effects) or of no consequence (i.e., some side effects). Integration of the extended phenotype concept with this categorization emphasizes that the microenvironment is not working together with the genetically altered cell as a partner in oncogenesis. Rather, it suggests that the most critical genetic instigators of oncogenesis alter the microenvironment exploitatively.

## Alternative conceptual frameworks

The barrier theory has been formulated in part by incorporating insights generated from other conceptual frameworks. It is therefore useful to compare and contrast these alternative frameworks, to allow for assessments of the value of each for future work on cancer.

Many of the arguments about oncogenic selection in the barrier theory are similar to one of the most influential paradigms of oncogenesis, which we refer to as the clonal diversification model. The central theme of the clonal diversification model is that oncogenic selection increases phenotypic and genotypic heterogeneity of cancer cells within and among tumors, with new mutant clones potentially arising from any cell in the tumor and with particular clones coming to prominence on the basis of superior reproductive capabilities (Fidler and Hart 1982). The clonal diversity model has had a lasting impact on the understanding of cancer; it still serves as a basis for modern assessments of oncogenesis (Greaves and Maley 2012) and has been supported by recent studies of genetic variation and cancer evolution (Nik-Zainal et al. 2012; Welch et al. 2012). Principles of evolution have been integrated into the clonal diversity model through consideration of the action of natural selection on cancer control mechanisms and the action of oncogenic selection on the clonal variants during oncogenesis (DeGregori 2011; Greaves and Maley 2012).

One way in which the barrier theory differs from the clonal diversification model as it was originally conceived (Fidler and Hart 1982) involves the role of heterogeneity in tumor stability. The clonal diversification model attributed stability to the genetic and phenotypic heterogeneity of the tumor (Fidler and Hart 1982). The barrier theory, in contrast, views this heterogeneity in the context of the many regulatory processes that are in place in normal cells as the result of natural selection. The large number of normal regulatory processes generates the potential for many possible dysregulations that can be favored by oncogenic selection. The barrier theory does not ascribe a role or function to heterogeneity. It interprets any apparent stability of tumors as a residual effect of the many regulatory processes that were present prior to oncogenesis and emphasizes that the multi-faceted regulation of normal cells is being eroded by oncogenic selection.

The stem cell theory of cancer provides another influential conceptual framework. In contrast with the clonal diversification model, the central tenet of the stem cell theory suggests that oncogenesis is driven by a small subset of dysregulated cells within the tumor. In its most dichotomous form, the cancer stem cell theory divides cancer cells into two categories: cancer stem cells and cells that are not cancer stem cells. This division stands in marked contrast to the heterogeneity expected from the clonal diversification model.

Like interpretations of microenvironmental influences on oncogenesis, aspects of the stem cell theory have been developed by analogy with tissue development and organogenesis (Clarke et al. 2006). Its basic premise is that a small, distinct subpopulation of the cells in a tumor – those possessing attributes of stem cells – is responsible for generating the tumor in its primary and metastatic sites.

A workshop of experts convened by the American Association for Cancer Research was charged with ‘evaluating data suggesting that cancers develop from a small subset of cells with self-renewal properties analogous to organ stem cells’ (Clarke et al. 2006; page 9339). In the workshop summary, a cancer stem cell was defined as ‘A cell within a tumor that possesses the capacity to self-renew and to cause the heterogeneous lineages of cancer cells that comprise the tumor’ (Clarke et al. 2006). These authors emphasized that cancer stem cells could be derived from differentiated cells that have acquired stem cell like characteristics or from actual stem cells (Clarke et al. 2006). For convenience and clarity, we will refer to the former as ‘cancer stem-like cells’ and the latter as ‘cancerous stem cells’.

These two origins are associated with different presumptions about the capacities of stem cells in cancer. The argument for cancerous stem cells (Clarke and Fuller 2006) is consistent with the mechanisms and principles of oncogenic selection that we describe in the barrier theory, once the barrier of asymmetric division has been compromised. By increasing the production of non-stem cells through an increase in symmetric division, cancerous stem cells could comprise a minority of tumor cells (Clarke and Fuller 2006); however, if the non-stem cells differentiate into nonreproducing cells, oncogenic selection will favor symmetric generation of cancerous stem cells, leading to a steadily increasing proportion of cancerous stem cells in the tumor.

The hypothetical derivation of cancer stem-like cells is the part of the theory that was constructed largely by analogy with normal tissue development. This analogy does not explain, however, why the differentiated cells that acquire stem cell-like characteristics would represent only a small proportion of the cells in the tumor. Such a reversion would require that both the cell and its microenvironment switch back to that of a stem cell; moreover, it is hard to envision why oncogenic selection would favor a cell that essentially discards most of its offspring as dead-end, differentiated cells. Rather, oncogenic selection should favor cells in accordance with their survival and reproductive advantages. Characteristics, such as immortalization of cells and release from cell cycle arrest, will make the predominant cancer cells similar to stem cells. Symmetric division and abrogation of apoptosis, however, will make them dissimilar from stem cells. If a cell has some similarities to stem cells it could be labeled ‘stem cell like’. But in the interests of clarity and parsimony, the barrier theory uses the specific phenotypic characteristics (e.g., resistance to apoptosis, telomerase activity, abrogated cell cycle arrest) rather than the stem cell metaphor.

In an effort to reconcile the problem of maintaining small numbers of cancer stem-like cells in tumors, Gupta et al. (2009) proposed that developmental transitions between epithelial and stem cell-like mesenchymal phenotypes could maintain the ratio of stem cell and differentiated phenotypes in a ‘dynamic equilibrium’. Such a dynamic equilibrium makes sense for tissue development and maintenance, but for it to apply to oncogenic selection, one would have to invoke frequency-dependent advantages. When viewed from the perspective of oncogenic selection, these sorts of attempts to rescue the importance of cancer stem-like cells take on the Kuhnian sense of a theory in crisis. We therefore expect that the part of the stem cell theory that advocates cancer stem-like cells will eventually fall by the wayside in competition with theories that are built more firmly on the foundation of oncogenic selection and emphasize a heterogeneous spectrum of variation.

Another conceptual framework, the hallmarks of cancer (Hanahan and Weinberg 2000), has been one of the most useful structures over the past dozen years for organizing the complex and ever-growing information on oncogenesis. The barrier theory of cancer presented here shares some features with the hallmarks of cancer, but differs distinctly in other aspects. Both frameworks attempt to understand oncogenesis by invoking a small number of underlying principles. The barrier theory focuses hierarchically on the causes of oncogenesis, whereas hallmarks can be considered to be an assessment of the defining features of cancer. In practice the importance of these features is related to their roles in oncogenesis, and this difference is less significant than it might seem at first glance.

A fundamental difference between the two frameworks involves the path of logic. The hallmarks have been formulated using an inductive approach – the state of current knowledge was examined to identify the key principles of oncogenesis. In contrast, the barrier theory uses the fundamental organizing principle of living systems – evolution by selection – as a first principle for understanding oncogenesis. Although inductive frameworks are often used to provide a scientific understanding of complicated systems, once inductive reasoning identifies key features of a system, they help generate and then tend to be replaced by frameworks that build on fundamental organizing principles.

Attempts to understand the bewildering complexity of living organisms illustrate this transition. Linnaeus provided one of the most significant early developments in this endeavor. His framework organized life systematically by categorizing living forms according to similarities and differences. By observing organisms in their natural environments, scientists such as Alexander von Humbolt used this system to identify the relationships between form and function in natural settings. Making use of these inductive frameworks, Darwin restructured our understanding of life based on the first principles of variation, survival, and reproduction to generate his theory of evolution by natural selection. By reorganizing our understanding of life on the basis of its generative process (i.e., selection acting on heritable variation), he provided a simple framework that accommodated the details known during his time as well as the vastly greater complexity of biological knowledge that has accumulated since then. This principle of Darwinian selection similarly offers a streamlined conceptual integration of the inductive insights into oncogenesis that are incorporated in the hallmarks of cancer.

Both the hallmarks and barrier frameworks emphasize some of the same features of oncogenesis, but do so for subtly different reasons. Both emphasize apoptosis. The hallmarks perspective implicates it because evasion of apoptosis has been shown to be important in oncogenesis. The barrier theory emphasizes apoptosis because differential survival and reproduction are the two components that determine the outcome of Darwinian selective processes, and apoptosis is the cell's mechanism for ending its survival during oncogenic selection and protects the genetic interests of the organism in the process of natural selection.

Similarly, both the hallmarks and the barrier frameworks identify limitless replicative potential during oncogenesis. Again, the hallmarks perspective implicates this attribute because it is a key feature of oncogenesis, whereas the barrier framework emphasizes it because continued reproduction provides a fundamental advantage in oncogenic selection.

Angiogenesis is considered one of the six hallmarks of cancer. Some evolutionary considerations have similarly assigned angiogenesis an importance on par with barriers such as the abrogation of apoptosis (e.g., DeGregori 2011; Sprouffske et al. 2011). Its place in the barrier theory is less significant because the lack of angiogenesis is not a barrier that needs to be compromised in order for oncogenesis to occur. The barrier theory therefore categorizes angiogenesis as an exacerbating cause rather than an essential cause.

The hallmarks of cancer contrast oncogenes and gain of function with tumor suppressors and loss of function. The barrier theory is agnostic on the inherent importance of these two categories except in so far as they prevent oncogenesis. Tumor suppressors take on a more central role than oncogenes in the barrier theory because tumor suppressors tend to be enforcers of barriers. Essential causes often abrogate tumor suppressor function, whereas mutations that convert proto-oncogenes into oncogenes tend to be exacerbating causes.

One disadvantage of the hallmarks framework is that it runs the risk of key concept proliferation, as the ever-growing base of knowledge implicates new processes in oncogenesis or as entirely different mechanisms are discovered. The initial formulation, for example, identifies two aspects of growth signals as hallmarks: self-sufficiency of growth signals and insensitivity of antigrowth signals. Sensitivity to growth signals and a lack of self-sufficiency of growth signals may contribute to a nondividing state or alter the rate of division, but it is the nondividing state (rather than the mechanism by which it is imposed) that is the barrier to cancer. Many different signaling mechanisms may generate a broad spectrum of effects on the rate of cell division and may thereby influence the ways in which dividing cells could become cancerous or enhance the severity of cancer, but these influences are exacerbating causes.

Hanahan and Weinberg (2011) have recently proposed two ‘emerging hallmarks’: reprogramming of energy metabolism and evasion of immune destruction. Although both processes contribute to oncogenesis, the barrier theory would not incorporate either as an essential cause unless it was shown that oncogenesis could not occur without genetically based alteration of the energy metabolism or immune evasion.

Cavallo et al. (2011) divided Hanahan and Weinberg's evasion-of-immune-destruction hallmark into three immunological hallmarks: the ‘ability to thrive in a chronically inflamed microenvironment’, evasion of immune recognition, and suppression of immune reactivity. Although each of these characteristics of oncogenesis is important, none could be incorporated into the barrier theory as an essential cause unless it was shown that oncogenesis could not proceed without a genetic alteration of the normal cell (by mutation or infection) to enact the characteristic. The indistinctness of terms such as ‘thrive’, the difficulty of immunological detection of precancerous cells (even without specific subterfuges), and the presence of cancer in people who are not immune suppressed all suggest that these immunological hallmarks may be exacerbating causes of cancer rather than essential causes.

Hanahan and Weinberg (2011) have also suggested that the influences of the tumor microenvironment need to be incorporated into the hallmarks of cancer. This proposition is undoubtedly true, but the complexity of the interactions among tumor cells and their microenvironments draws attention to the need for a framework that distills the essence of these interactions. We believe that the incorporation of the extended phenotype concept into the barrier theory does so in a way that is tightly integrated with the evolutionary principles of natural selection and oncogenic selection.

Hanahan and Weinberg (2011) have added two ‘enabling characteristics’: (i) tumor-promoting inflammation and (ii) genomic instability and mutation. The barrier theory incorporates the first of these as part of the extended phenotype and considers genomic instability and mutation to be two quite different aspects of oncogenesis. Mutation is the source of variation on which oncogenic selection acts, and genomic instability is a consequence of mutation as well as a cause. There are many regulations imposed on cellular processes by natural selection. The compromising of any one of them could provide a cell with a net growth advantage over more strictly regulated cells. The great diversity of mutations that can thus be favored during oncogenic selection apparently allows genomic instability to be favored during oncogenesis (e.g., through the spread of mutator phenotypes).

## Implications for treatment and prevention

The barrier theory emphasizes that cancer prevention will require a focus on essential causes. Treatments that interfere with exacerbating causes may be useful in ameliorating cancer but are unlikely to lead to cures or prevention. The barrier theory therefore suggests that prevention strategies will need a better understanding of infectious causation of cancer because pathogens are packets of multiple essential causes.

If essential and exacerbating causes are not distinguished, prospects for targeted therapies are dim because most potential targets are exacerbating causes, and cancers can escape from this sort of intervention – destruction of cells that rely on the exacerbating cause leaves behind cells that do not.

We have emphasized the difference between oncogenesis as a selective process and organogenesis as a guiding metaphor. If assessments of microenvironmental influences are structured by the metaphor of organogenesis (e.g., Egeblad et al. 2010) rather than the extended phenotype, strategies for controlling cancer could be guided to relatively ineffective interventions. Considering the role of normal cells in the tumor microenvironment, Egeblad et al. (2005) refer to fibroblasts as co-conspirators in oncogenesis. The extended phenotype perspective casts them instead as pawns – cells that are acting in accordance with direction from cancer cells. The difference may seem trivial, but the implications for intervention are not, because targeting a co-conspirator implies a more decisive intervention than targeting a pawn. The extended phenotype perspective does not yield optimism about interfering with angiogenesis or metalloproteases as strategies for combatting cancer. Indeed, the track record of innovative strategies based on inhibiting angiogenesis and metalloprotease activity has been disappointing relative to expectations of researchers whose assessments were not structured by the extended phenotype perspective. The analyses presented by Egeblad et al. (2005) steered clear of this trap because they focused on the cancerous cells, but the potential for misdirection exists if researchers become accustomed to thinking of microenvironmental constituents as more equal drivers rather than as aspects of the cancer cell's extended phenotype.

The stem cell theory has been attractive to researchers in part because, if valid, it offers hope for improving cancer treatments. Specifically, it suggests that the targeting of stem cells could remove the cells that are responsible for generating, maintaining and regenerating cancers (e.g., following chemotherapy) (Clarke and Fuller 2006; Tomasson 2009).

The focus on oncogenic selection raises grave doubts about the part of the stem cell theory that posits reversion of differentiated cells to cancer stem cells. These concerns suggest that hopes for preventive or therapeutic benefits from the stem cell theory and investment in these options should be tempered by the aspects of the theory that are drawn from analogy.

A major difference between the stem cell theory and the barrier theory is evident in the context of new oncogenic selective pressures such as those imposed by cancer therapy, resource restriction in the microenvironment, and within-tumor cell competition arising from new mutations. The stem cell theory accommodates the possibility that oncogenic selection could cause evolutionary changes in the stem cell lineages, but treats these changes as a moving target rather than as diversification of the stem cell lineage into many different lineages (Clarke et al. 2006). The barrier theory (like the clonal diversification model) emphasizes that oncogenic selection will lead to genetic and phenotypic diversity because new variants overgrow but are unlikely to replace completely other variants.

This distinction between the stem cell and barrier theories therefore has important implications for interventions. Even if all could agree that targeting stem-like properties for treatment might increase the likelihood of eradicating the most imminently dangerous cells, if the remaining cell population has the capacity to spin off new mutants at any time with abilities to adapt to the current environment (e.g., through drug resistance, see Gillies et al. 2012), then focusing on a small portion of the tumor cells that are deemed cancer stem cells may prove to be demoralizing and harmful.

The barrier theory emphasizes the need to identify barriers that are compromised in particular cancers. Countering a barrier-destroying mutation will be more potent therapeutically than countering a restraint-removing mutation. If an intervention targets more than one barrier, it can be even more effective. Chronic myeloid leukemia offers an illustration. The fusion protein that characterizes chronic myeloid leukemia compromises cell adhesion and apoptosis in addition to accelerating growth rates (Deininger et al. 2000). The therapeutic silencing of this protein has been one of the most successful examples of targeted therapy.

The tumor viruses mentioned above are packages of multiple essential causes because they abrogate four barriers simultaneously (Ewald and Swain Ewald 2012). Moreover, infectious agents generate nonhuman targets for interventions, which therefore can be attacked with fewer adverse effects than tend to occur when human molecules are targeted. In this context, it is not surprising that control of infectious causes of cancer – through vaccines against HPV and HBV, prevention of transmission of HBV, HCV, and *Helicobacter pylori*, antibiotic treatment of *Helicobacter pylori* – ranks among the most successful actions against cancer. The barrier theory emphasizes that concerted efforts to discover the full scope of infectious causation of cancer could be one of the most effective investments in the efforts to control cancer.

Drawing on insights from current conceptual frameworks for oncogenesis and applying principles of Darwinian selection, we formulated the barrier theory of oncogenesis to provide a simple, versatile evolutionary framework for understanding cancer. We hope that this construct will provide the basis for a more thorough evolutionary theory of cancer. Our general goal is to provide an approach based on first principles with sufficient flexibility to accommodate current knowledge about oncogenesis as well as knowledge that will be acquired in the future. Our practical goal is to help identify interventions that offer particularly good prospects for preventing and treating cancer.
